# Multivendor comparison study of artificial intelligence software for automated fracture detection in paediatric patients

**DOI:** 10.1007/s00247-025-06461-6

**Published:** 2025-11-18

**Authors:** Irmhild Altmann-Schneider, Léa S. Geiger, Christian J. Kellenberger, Fraser Callaghan, Michelle Seiler

**Affiliations:** 1 Department of Diagnostic Imaging, University Children’s Hospital Zurich, Lenggstrasse 30, Zurich, 8008 Switzerland; 2 Children’s Research Centre, University Children’s Hospital Zurich, Zurich, Switzerland; 3 Paediatric Emergency Department, University Children’s Hospital Zurich, Zurich, Switzerland; 4 Centre for MR-Research, University Children’s Hospital Zurich, Zurich, Switzerland

**Keywords:** Artificial intelligence, Emergency service, Fractures, Joint dislocations, Software

## Abstract

**Background:**

Evidence for artificial intelligence (AI)-assisted paediatric fracture detection is limited. External validation and comparison of AI software are required before reliable use in clinical practice.

**Objective:**

To evaluate and compare the performance of three commercially available AI software for detecting posttraumatic findings in paediatric patients.

**Materials and methods:**

This retrospective study assessed three AI software using radiographs of children aged 2–17 years who presented to the emergency department after trauma. Radiographs of the lower leg, forearm, and elbow were included between January 2014 and January 2024 (lower leg), March 2022 and January 2024 (forearm), and July 2019 and January 2024 (elbow). Sensitivity, specificity, positive predictive value (PPV), and negative predictive value (NPV) were calculated for fractures, effusions, and dislocations.

**Results:**

A total of 3,013 patients with 3,414 radiographs were included: 1,074 lower leg (mean age 6.6 years), 1,142 forearm (7.4 years), and 1,198 elbow (7.5 years). All AI tools demonstrated high performance for lower leg and forearm radiographs, with sensitivity of 88.4–94.7%, specificity 93.6–99.2%, PPV 94.5–99.2%, and NPV 91.6–95.6%. In contrast, performance for elbow radiographs was reduced (sensitivity 72.8–91.6%, specificity 80.3–98.7%), with the lowest PPV of 86.1% and NPV of 79.5%. Sensitivity was notably reduced for specific paediatric fracture types, elbow effusions (posterior fat pad sign 40.6–82.3%), and dislocations (54.2–93.8%), with significant differences between AI software.

**Conclusions:**

AI tools show promise for paediatric fracture detection, particularly in lower leg and forearm radiographs. Awareness of their limitations is essential for safe clinical use.

**Graphical abstract:**

**Figure:**
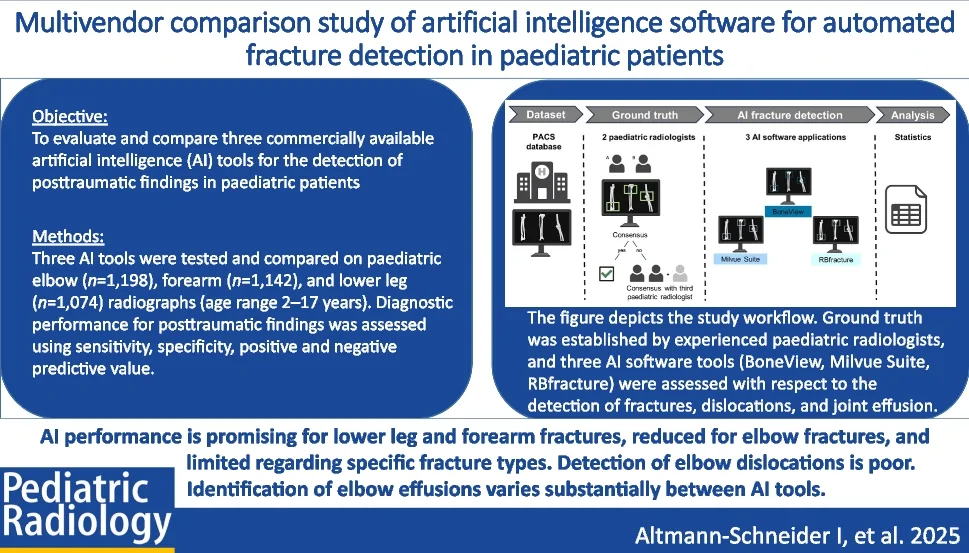

**Supplementary Information:**

The online version contains supplementary material available at https://doi.org/10.1007/s00247-025-06461-6.

## Introduction

Fractures are a leading cause of visits to paediatric emergency departments (EDs) [1]. Their detection on radiographs is more challenging than in adults due to the dynamic nature of the growing skeleton, developmental variations in bone maturation with age-dependent fracture types and injury patterns [2, 3]. Elbow fractures are particularly difficult to diagnose, as they are often occult and may only be inferred from indirect signs such as joint effusion [4–6].

Numerous AI-based tools have been developed to assist in fracture detection, aiming to improve diagnostic accuracy for front-line clinicians interpreting radiographs in trauma settings. However, most have been trained and validated on adult patients, with limited evidence for paediatric fractures [7–9]. Existing studies on the use in paediatric patients vary widely in age groups, anatomical regions, and sample sizes [10, 11]. Large trials addressing specific regions, fracture types, and indirect fracture signs remain scarce [12]. Moreover, only a few AI tools have undergone external validation [11], and when tested, performance often declined [9]. Comparative trials assessing diagnostic accuracy, detection of indirect fracture signs, and joint dislocations in paediatric patients are lacking, leaving physicians with little guidance in selecting the most appropriate AI software.

Our study aimed to address these gaps by evaluating and comparing three (of four) commercially available AI tools. Performance was assessed on a large dataset of three common paediatric fracture sites: the lower leg, forearm, and elbow, including distinct fracture types, indirect fracture signs in the elbow, and joint dislocations.

## Methods

### Ethical approval

This study was approved by the cantonal research ethics committee according to the Code of Ethics of the World Medical Association (Declaration of Helsinki) (BASEC-Nr. 2022-00816).

### Data acquisition and patients

**Fig. 1:**
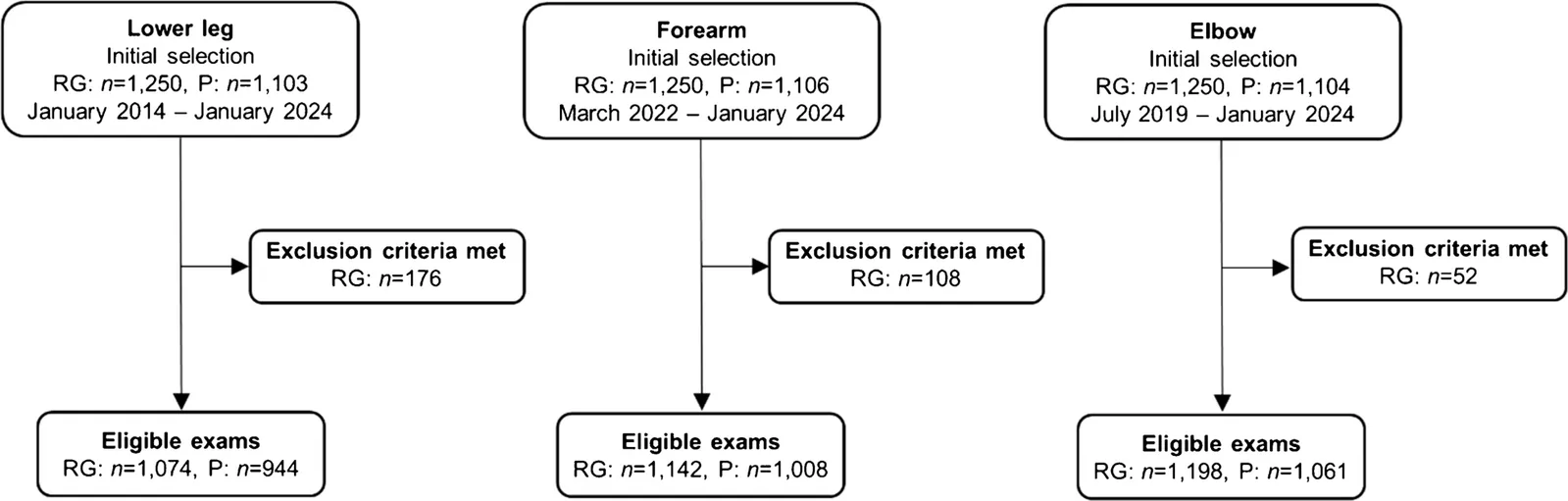
Flow chart depicting the process of inclusion and exclusion of radiographs. *n* number,* P* patients, *RG* radiographs

Consecutive radiographs of patients aged 2–17 years presenting to the ED with acute trauma were retrospectively included from January 2014 until January 2024 (lower leg), March 2022 until January 2024 (forearm), and July 2019 until January 2024 (elbow). The initial selection included 1,250 radiographs per region from 1,103 (lower leg), 1,106 (forearm), and 1,104 (elbow) patients, of which 176 (lower leg), 108 (forearm), and 52 (elbow) were excluded (Fig. 1).

This target was based on a sample size estimation using a binomial model (*n*=4*p*(1-*p*)/*d*2; *d*=0.05), which indicated a minimum of 336 radiographs with fractures (sensitivity 70%) and 144 without fractures (specificity 90%). To account for age variability (2–17 years) and fracture types, we aimed for approximately 1,000 radiographs per region and increased this number to 1,250 to account for exclusions and preserve power. Inclusion criteria were radiographs performed on the day of ED attendance of either the forearm, lower leg, or elbow in at least two projections (frontal/lateral following our institutional protocol), with image quality verified by a paediatric radiologist prior to inclusion. Exclusion criteria included failed AI analysis, single-projection radiographs, non-trauma imaging, subacute trauma, fractures highly specific for non-accidental injury, and prior study inclusion [12].

Paired bones in the lower leg and forearm, and multiple posttraumatic findings, were analysed separately as distinct cases. Radiographs of insufficient quality for assessing elbow effusion were excluded from the analysis.

Radiographs were acquired on two radiographic machines (Philips, Best, The Netherlands; Siemens Healthcare, Erlangen, Germany).

### Reference standard and readers

**Fig. 2:**
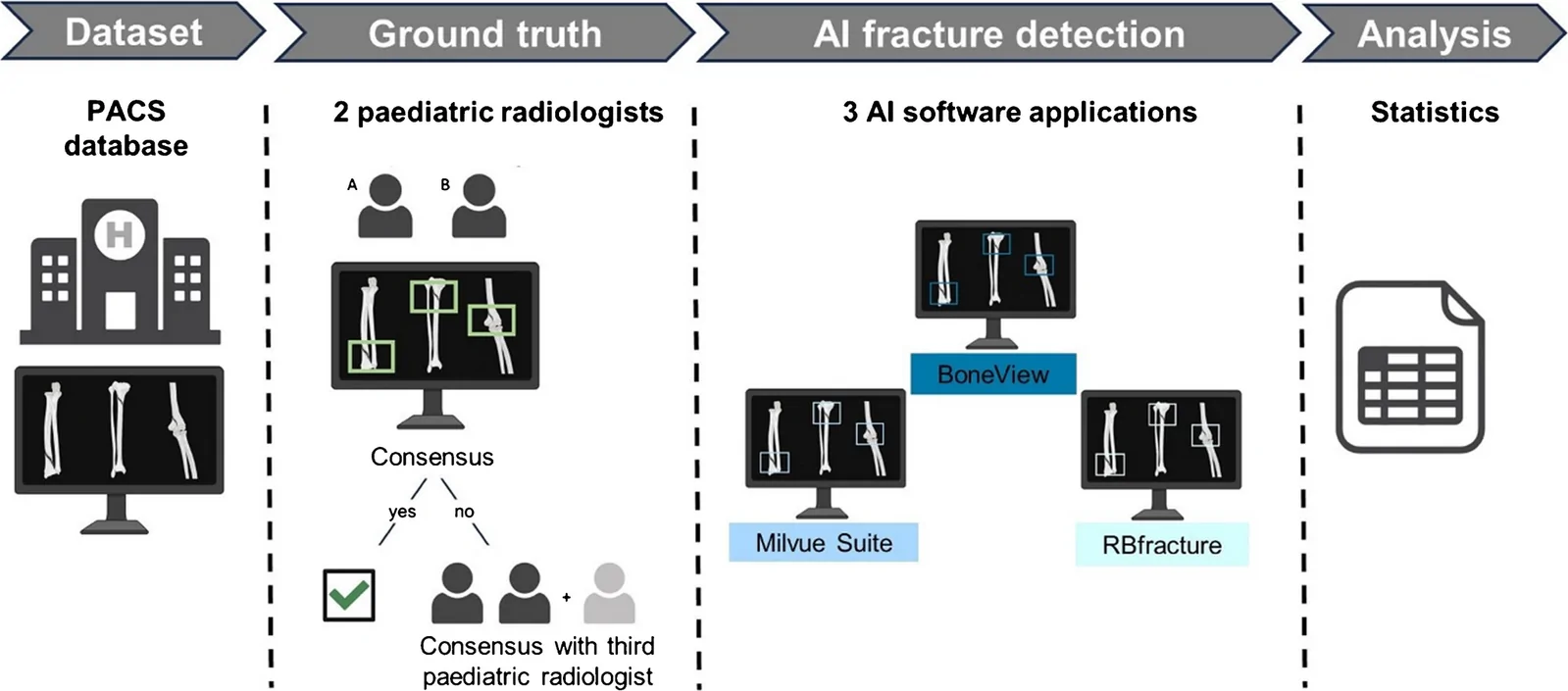
Study design and workflow. *AI* artificial intelligence, *PACS* picture archiving and communication system

Radiographs were initially reported by an attending paediatric radiologist (varying levels of experience). A certified paediatric radiologist (6 years’ experience) performed a second reading with access to clinical data and follow-up imaging. Discrepancies were resolved by consensus with a third paediatric radiologist (>5 years’ experience). This consensus diagnosis served as the reference standard. Fracture types were further analysed when ≥10 cases were available. Images were reviewed on Sectra workstations (IDS7, Sectra AB, Linköping, Sweden). Study design and workflow are illustrated in Fig. 2.

### Artificial intelligence software applications

Of the four CE (Conformité Européenne)-certified and/or FDA (Food and Drug Administration)-approved AI tools for paediatric fracture detection, three (BoneView (BV), RBfracture (RBf), and Milvue Suite (MS)) participated (Table 1). One vendor (Rayvolve, AZmed, Paris, France) declined.

**Table 1:** Overview of artificial intelligence software characteristics

Software	Company	Certification	Market presence	Approval	Output with confidence score
BoneView	Gleamer, Paris, France	CE-certified FDA-approved	03/2020	>2 years	Fractures only
Milvue Suite	Milvue, Paris, France	CE-certified	02/2020	No limitation	Fractures, dislocations, effusion
RBfracture	Radiobotics Copenhagen, Denmark	CE-certified	06/2022	>2 years (fractures) >15 years (dislocation and effusion)	Fractures, dislocation, effusion

*CE* conformité européenne, *FDA* Food and Drug Administration

Vendors provided the latest trial version of their AI, with no modifications allowed. Analyses were performed concurrently in September and October 2024. Our institution has used BV under licence since 2023. For previously analysed cases, BV analyses were repeated to verify consistency.

*BoneView™* (Gleamer, Paris, France) is a commercially available AI-based computer-aided diagnosis tool. It employs a deep convolutional neural network built on the Detectron2 two-stage object detection framework. The algorithm applies three operating points: *negative* (confidence <50%), *doubt* (confidence 50–90%), and *positive* (confidence >90%). Operating points were defined from the free-response operating characteristic curve of the development test set to balance high sensitivity and specificity. For effusion and dislocation assessment, only negative and positive outputs are used. The algorithm was trained on >300,000 radiographs from over 60 radiology departments (January 2011–May 2023), randomly split into 70% training, 10% validation, and 20% internal test sets without overlap.

*Milvue Suite™* (Milvue, Paris, France), with its *TechCare Alert/Kids* configuration, is a CE-marked Class IIa medical device; a derived version (TechCare Trauma, without dislocation detection) is FDA-cleared. Its deep learning algorithms were trained on 121,808 annotated radiographic sets from French radiology centres, covering all anatomical regions. Annotations were produced by consensus of qualified radiologists. Testing used 80,938 consensus-annotated radiographs with balanced demographics. Outputs are classified into three statuses: *no* (no suspicious area), *doubt* (confidence >0.10, high-sensitivity point), and *yes* (confidence >0.90, high-specificity point).

*RBfracture™* v. 2.2 (Radiobotics, Copenhagen, Denmark) is a CE-marked Class IIa medical device. It uses a Deep Neural Network built on the Detectron object detection framework, specifically tailored to optimize fracture detection in radiographs. The model was trained on >450,000 radiographs from >160,000 patients across >1,300 radiology departments worldwide. Outputs vary by diagnostic confidence: detections with solid bounding boxes indicate high confidence (PPV ≥95%), while dashed boxes represent lower certainty (NPV 95%). Thresholds were optimized using an internal tuning dataset with natural prevalence to achieve clinically meaningful performance.

### Statistical analyses

Statistical analyses were performed using R Studio (R version 4.4.2 (2024-10−31)). Sensitivity, specificity, PPV, and NPV with 95% confidence intervals (CIs) were calculated for fractures, dislocations, and elbow effusions. Anterior and posterior elbow effusions (aka fat pad signs) were assessed separately. Each dataset was analysed twice: once considering doubt cases as positive and once as negative. Unless otherwise stated, *doubt* was interpreted as positive. Comparisons between AI tools were performed using chi-square, or where appropriate, Fisher’s exact test. Post hoc pairwise analyses used proportion tests or Fisher’s exact test, with Holm correction for multiple testing. A significance threshold of *P*<0.05 was applied. As MS and RBf filter joint views, elbow effusions and dislocations were not consistently assessed. Analyses restricted to cases available from all applications were repeated and reported separately.

## Results

A total of 3,414 patients were included. Mean age was 7.4±3.6 years for forearm (54% male), 7.5±3.5 years for elbow (51% male), and 6.6±4.3 years for lower leg radiographs (57% male). Detailed population characteristics are presented in Table 2.

**Table 2:** Patient characteristics

Patient characteristic	Lower leg	Forearm	Elbow
Mean age±SD	6.6±4.3	7.4±3.6	7.5±3.5
Sex			
Females, *n*	461	522	584
Males, *n*	613	620	615

*n* number, *SD* standard deviation

### Lower leg

A total of 1,074 radiographs (2,192 cases) were included, comprising 680 fractures: 502 (74%) tibial, 175 (26%) fibular, and 3 (0.4%) femoral fractures (Table 3), corresponding to frequencies of 47%, 16%, and 0.3%.

**Table 3:** Radiograph characteristics per anatomical region

	Lower leg	Forearm	Elbow
Radiographs, *n*	1,074	1,142	1,198
Cases, *n*	2,192	2,424	1,357
Radius/ulna, *n*		1,172/1,164	180/98
Humerus, *n*		88	593
Fibula/tibia, *n*	1,089/1,098		
Femur, *n*	5		
Fractures, *n*	680	1,345	654
Radius/ulna, *n*		768/550	116/69
Humerus, *n*		27	469
Fibula/tibia, *n*	175/502		
Femur, *n*	3		
Dislocations, *n*		23	48
Effusion, *n*			716
Anterior fat pad sign, *n*			126
Posterior fat pad sign, *n*			70
Both, *n*			520

*n* number

Sensitivity and PPV exceeded 90% for all AI tools, except RBf (sensitivity 88.4%). Specificity and NPV were >95% for all tools. When doubt cases were classified as negative, sensitivity declined for all AI, most notably RBf (−8 percentage points), with corresponding decreases in NPV and increases in specificity and PPV (Table 4).

**Table 4:** Lower leg radiographs

Parameter	Doubt=fracture	Doubt=no fracture
*BoneView*	Total number of doubt cases: 66	
Sensitivity (95% CI)	91.8 (89.5–93.6)	85.9 (83.1–88.3)
Specificity (95% CI)	97.9 (97.0–98.5)	99.6 (99.1–99.8)
Positive predictive value (95% CI)	95.1 (93.2–96.5)	99.0 (97.8–99.5)
Negative predictive value (95% CI)	96.4 (95.3–97.2)	94.0 (92.7–95.1)
*Milvue Suite*	Total number of doubt cases: 7	
Sensitivity (95% CI)	90.0 (87.5–92.0)	89.7 (87.2–91.8)
Specificity (95% CI)	98.8 (98.1–99.3)	99.1 (98.5–99.5)
Positive predictive value (95% CI)	97.1 (95.5–98.2)	97.9 (96.5–98.8)
Negative predictive value (95% CI)	95.6 (94.5–96.6)	95.5 (94.4–96.5)
*RBfracture*	Total number of doubt cases: 78	
Sensitivity (95% CI)	88.4 (85.8–90.1)	80.3 (77.1–83.1)
Specificity (95% CI)	97.7 (96.8–98.3)	99.2 (98.6–99.6)
Positive predictive value (95% CI)	94.5 (92.4–96.0)	97.8 (96.3–98.8)
Negative predictive value (95% CI)	94.9 (93.7–95.9)	91.8 (90.4–93.0)

*CI* confidence interval

Comparative analyses showed no significant differences between AI (Supplementary Material 1).

RBf detected most doubt cases (BV 66; MS 7; RBf 78), with > two-thirds confirmed as true positives (BV 40; MS 2; RBf, 55). Accordingly, RBf achieved the highest PPV (95% CI): BV 60.6% (48.5–71.5), MS 28.6% (8.7–64.1), RBf 70.5% (59.6–79.5).

The most frequent fracture types, oblique tibial shaft, proximal tibial buckle, and toddler’s fractures, accounted for 45% of all fractures. Detection rates exceeded 90% for most types, except proximal tibial buckle, toddler’s, both proximal and distal tibial Salter-Harris II, and distal tibial Salter-Harris IV fractures. Reduced detection was also noted for RBf in oblique diaphyseal and proximal fibular buckle fractures, and for BV in complete proximal metaphyseal fibular fractures (Supplementary Material 2).

### Forearm

A total of 1,142 radiographs (2,424 cases) were included, comprising 1,345 fractures: 27 (2%) humeral, 768 (57%) radial, and 550 (41%) ulnar fractures (Table 3), corresponding to frequencies of 2%, 67%, and 48%.

Sensitivity and PPV were ≥92% for all AI tools, while specificity and NPV exceeded 91%. When doubt cases were considered negative, sensitivity declined for all AI, most notably RBf (−6 percentage points), with corresponding reductions in NPV and increases in specificity and PPV (Table 5).

**Table 5:** Forearm radiographs

Parameter	Doubt=fracture	Doubt=no fracture
*BoneView*	Total number of doubt cases: 106	
Sensitivity (95% CI)	93.5 (92.1–94.7)	90.0 (88.3–91.5)
Specificity (95% CI)	93.6 (92.0–94.9)	99.1 (98.3–99.5)
Positive predictive value (95% CI)	94.8 (93.5–95.9)	99.2 (98.5–99.6)
Negative predictive value (95% CI)	92.1 (90.3–93.5)	88.9 (87.0–90.5)
*Milvue Suite*	Total number of doubt cases: 31	
Sensitivity (95% CI)	94.7 (93.4–95.8)	93.4 (91.9–94.6)
Specificity (95% CI)	96.4 (95.1–97.3)	97.6 (96.5–98.4)
Positive predictive value (95% CI)	97.0 (96.0–97.8)	98.0 (97.1–98.6)
Negative predictive value (95% CI)	93.6 (92.0–94.9)	92.2 (90.5–93.6)
*RBfracture*	Total number of doubt cases: 111	
Sensitivity (95% CI)	92.9 (91.4–94.1)	86.6 (84.7–88.3)
Specificity (95% CI)	96.7 (95.4–97.6)	99.2 (98.4–99.6)
Positive predictive value (95% CI)	97.2 (96.1–98.0)	99.2 (98.5–99.6)
Negative predictive value (95% CI)	91.6 (89.9–93.2)	85.6 (83.5–87.4)

*CI* confidence interval

MS and RBf demonstrated significantly higher specificity than BV (Supplementary Material 3).

RBf and BV detected roughly three times more doubt cases than MS (BV 106; MS 31; RBf 111), with RBf yielding the highest true-positive rate (BV 47; MS 18; RBf 84). Accordingly, the PPV (95% CI) of doubt cases was highest for RBf: BV 44.3% (35.2–53.8), MS 58.1% (40.8–73.6), and RBf 75.7% (66.9–82.7).

The most common fracture types were distal metaphyseal buckle fractures of the radius and ulna, accounting for ~40% of all fractures. Detection rates exceeded 95% for most types, except distal ulnar buckle fractures, ulnar styloid avulsions, proximal radial buckle fractures, complete proximal metaphyseal ulnar fractures, and proximal radial Salter-Harris II fractures. Radial and ulnar bowing fractures were not consistently detected by all AI tools (Supplementary Material 4).

The dataset included 23 elbow dislocations (14 Monteggia injuries) and one distal ulnar dislocation (Galeazzi fracture). Only 373 cases were assessed for dislocation by MS. RBf output was missing in two cases. BV and RBf showed low sensitivity and PPV (95% CI): BV 21.7% (9.7–42.0)/45.5% (21.3–72.0), RBf 43.5% (25.6–63.2)/40.0% (23.4–59.3), with specificity and NPV >99% for both. A total of 21 false-positive dislocations (wrist: 10, elbow: 11) were detected (elbow: BV 4, MS 3, RBf 6; wrist: BV 1, MS 5, RBf 8). Repeating analyses for the 373 cases assessed by MS improved BV and RBf performance. MS reached 100% sensitivity but with limited PPV (38.5%) (Table 6). Comparative analysis within this subset revealed no significant differences.

**Table 6:** Forearm radiographs – dislocations

Parameter	BoneView	Milvue Suite	RBfracture
		Doubt=dislocations	Doubt=dislocations
Sensitivity (%) (95% CI)	66.0 (23.1–88.2)	100 (61.0–100)	80.0 (37.6–96.4)
Specificity (%) (95% CI)	98.6 (96.9–99.4)	96.7 (94.4–98.1)	98.6 (96.9–99.4)
Positive predictive value (95% CI)	37.5 (13.7–69.4)	33.3 (16.3–56.3)	44.4 (18.9–73.3)
Negative predictive value (95% CI)	99.5 (98.0–99.9)	100 (98.9–100)	99.7 (98.5–100)

*CI* confidence interval

### Elbow

A total of 1,198 radiographs (1,357 cases) were included, comprising 654 fractures: 469 (72%) humeral, 116 (18%) radial, and 69 (11%) ulnar fractures (Table 3), corresponding to frequencies of 39%, 10%, and 6%.

Sensitivity ranged from 87.5% (RBf) to 91.6% (MS), slightly lower than for forearm radiographs, while specificity varied between 80.3% (BV) and 86.8% (RBf). When doubt cases were classified as false positives, sensitivity and NPV decreased, while specificity and PPV increased (Table 7).

**Table 7:** Elbow radiographs

Parameter	Doubt=fracture	Doubt=no fracture
*BoneView*	Total number of doubt cases: 157	
Sensitivity (95% CI)	90.7 (88.2–92.7)	83.9 (80.9–86.6)
Specificity (95% CI)	80.3 (77.1–83.0)	96.4 (94.8–97.6)
Positive predictive value (95% CI)	81.1 (78.1–83.8)	95.6 (93.6–97.0)
Negative predictive value (95% CI)	90.2 (87.6–92.3)	86.5 (83.9–88.7)
*Milvue Suite*	Total number of doubt cases: 64	
Sensitivity (95% CI)	91.6 (89.2–93.5)	89.8 (87.2–91.9)
Specificity (95% CI)	82.4 (79.4–85.1)	89.9 (87.4–91.9)
Positive predictive value (95% CI)	83.0 (80.0–85.5)	89.2 (86.6–91.4)
Negative predictive value (95% CI)	91.3 (88.8–93.3)	90.4 (88.8–92.3)
*RBfracture*	Total number of doubt cases: 179	
Sensitivity (95% CI)	87.5 (84.7–89.8)	72.8 (69.3–76.1)
Specificity (95% CI)	86.8 (84.1–89.1)	98.7 (97.6–99.3)
Positive predictive value (95% CI)	86.1 (83.3–88.6)	98.1 (96.5–99.0)
Negative predictive value (95% CI)	88.1 (85.4–90.3)	79.5 (76.7–82.0)

*CI* confidence interval

RBf demonstrated significantly better detection of negative cases than BV (Supplementary Material 5).

RBf detected most doubt cases (BV 157; MS 64; RBf 179), with >50% confirmed as true positives (BV 44; MS 12; RBf 96), reflected in the PPV (95% CI): BV 28.0% (21.6–35.5), MS 18.8% (11.1–30.0), RBf 53.6% (46.3–60.8).

Complete supracondylar humerus fractures accounted for 45% of all fractures (Supplementary Material 6), with detection rates >98% for all AI. Supracondylar buckle fractures were detected >90% only by BV (RBf 57.7%). Radial condyle fractures were detected with rates of 89.2% (MS) to 95.4% (BV). Limited performance was observed for medial epicondyle avulsions (all AI tools), complete olecranon (BV, RBf), and proximal metaphyseal radial buckle fractures (MS, RBf).

Detection rates for proximal metaphyseal buckle fractures were higher on elbow (91.9%) compared to forearm (80.8%) radiographs for BV, despite comparable fracture frequency (32 versus 26). The same applied to proximal radial Salter-Harris II fractures for all AI, with the largest difference for BV (95.5% versus 83.3%). However, fracture frequency was considerably lower on forearm radiographs (12 versus 44). Detection of proximal complete metaphyseal ulna fractures differed most for MS (90% versus 83.3%), again with a lower frequency on forearm radiographs (18 versus 50).

Among 48 dislocations, BV correctly identified 26, MS 31, and RBf 45. Only RBf exceeded 93% sensitivity, while BV and MS achieved 54.2% and 64.4%, respectively. Specificity was high for all AI (BV 100%). PPV was highest for BV but low for RBf (53.6%), whereas NPVs were high for all tools (Table 8).

**Table 8:** Elbow dislocations

Parameter	Doubt=dislocation	Doubt=no dislocation
*BoneView*		
Sensitivity (95% CI)	54.2 (40.3–67.4)	n.a
Specificity (95% CI)	100 (99.7–100)	n.a
Positive predictive value (95% CI)	100 (87.1–100)	n.a
Negative predictive value (95% CI)	98.3 (97.5–98.9)	n.a
*Milvue Suite*		
Sensitivity (95% CI)	64.6 (50.4–76.6)	62.5 (48.3–74.8)
Specificity (95% CI)	99.2 (98.5–99.5)	99.9 (99.6–100)
Positive predictive value (95% CI)	73.8 (58.9–84.7)	96.8 (83.8–99.4)
Negative predictive value (95% CI)	98.7 (97.8–99.2)	98.6 (97.9–99.1)
*RBfracture*		
Sensitivity (95% CI)	93.8 (83.2–97.9)	79.6 (66.4–88.5)
Specificity (95% CI)	97.0 (95.9–97.8)	99.3 (98.7–99.6)
Positive predictive value (95% CI)	53.6 (43.0–63.9)	81.3 (68.1–89.8)
Negative predictive value (95% CI)	99.8 (99.3–99.9)	99.2 (98.6–99.6)

*CI* confidence interval, *n.a* not applicable

When doubt cases were treated as negative, MS sensitivity remained stable, and RBf showed low PPV (18.9%, seven true positives/37 doubt cases). Overall, RBf performed best for identifying positive cases, and BV for negative cases (Supplementary Material 5).

Elbow effusion was present in 716 radiographs: 126 with isolated anterior fat pad signs, 70 with isolated posterior fat pad signs, and 520 with both (Table 3), with an overall frequency of 60%. Sensitivity for anterior fat pad detection was ~84% for all AI, with specificity >92% for BV and MS, and 84% for RBf. PPV was highest for MS (98.4%), followed by BV (93.5%) and RBf (87.7%). NPVs ranged from 80.1% (RBf) to 82.6% (MS). Sensitivity for posterior fat pad detection ranged from 82.3% (MS) to 40.6% (BV), with high specificity for all tools (Table 9).

**Table 9:** Elbow effusion

Parameter	Anterior effusion		Posterior effusion	
	Doubt=effusion	Doubt=no effusion	Doubt=effusion	Doubt=no effusion
*BoneView*				
Sensitivity (95% CI)	84.3 (81.3–86.9)	n.a	40.6 (36.7–44.6.7.6)	n.a
Specificity (95% CI)	92.2 (89.4–94.2)	n.a	97.8 (96.1–98.7.1.7)	n.a
Positive predictive value (95% CI)	93.5 (91.2–95.2)	n.a	95.2 (91.8–97.2.8.2)	n.a
Negative predictive value (95% CI)	81.6 (78.1–84.6)	n.a	60.2 (56.9–63.4.9.4)	n.a
*Milvue Suite*				
Sensitivity (95% CI)	84.5 (81.5–87.1)	79.2 (75.9–82.2)	82.3 (79.1–85.2.1.2)	74.2 (70.5–77.6)
Specificity (95% CI)	98.1 (96.5–99.0)	99.0 (97.6–99.6)	99.1 (97.9–99.6.9.6)	99.6 (99.7–100)
Positive predictive value (95% CI)	98.4 (96.9–99.1)	99.0 (97.8–99.6)	99.0 (97.6–99.6.6.6)	99.5 (98.4–100)
Negative predictive value (95% CI)	82.6 (79.3–85.5)	78.2 (74.7–81.3)	83.8 (80.7–86.4.7.4)	78.0 (74.8–80.9)
*RBfracture*				
Sensitivity (95% CI)	84.5 (81.5–87.2.5.2)	73.8 (70.3–77.1)	73.6 (69.9–77.1.9.1)	64.3 (60.3–68.2)
Specificity (95% CI)	84.0 (80.5–87.1.5.1)	95.5 (93.3–97.1)	99.6 (98.7–99.9.7.9)	99.8 (99.0–100)
Positive predictive value (95% CI)	87.7 (84.9–90.1.9.1)	95.7 (93.5–97.2)	99.5 (98.3–99.9.3.9)	99.7 (98.5–100)
Negative predictive value (95% CI)	80.1 (76.4–83.4.4.4)	73.0 (69.4–76.4)	78.0 (74.8–81.0.8.0)	72.5 (69.1–75.6)

*CI* confidence interval, *n.a.* not applicable

A high proportion of doubt cases was true positives, resulting in high PPVs for both anterior and posterior fat pad signs (Supplementary Material 7).

RBf did not assess 27 radiographs for effusion. Exclusion of these cases did not significantly affect the results.

MS detected the posterior fat pad more accurately than other AI, whereas RBf demonstrated a higher specificity compared to BV. MS was most accurate in detecting the anterior fat pad sign (Supplementary Material 5).

## Discussion

We evaluated the diagnostic performance of three CE-certified AI tools for paediatric fracture detection in three anatomical regions with high fracture prevalence: the lower leg, forearm, and elbow. Performance was high for lower leg and forearm fractures, but reduced for elbow fractures. All AI tools demonstrated limitations in identifying specific fracture types. Detection of (elbow) dislocations was generally poor, except for one AI system, which nevertheless showed a low PPV. Detection of elbow joint effusion was moderate, with substantial variability between AI tools, particularly in identifying the posterior fat pad sign.

### Lower leg

Diagnostic performance for fracture detection was high across all AI tools, consistent with prior literature. Although paediatric lower leg radiographs have not been exclusively studied, reported sensitivities and specificities for lower extremity fractures range from 73–100% and 71–100%, respectively [13–17]. In our study, performance varied by fracture type and was notably reduced for toddler’s fractures, proximal metaphyseal buckle fractures, proximal and distal Salter-Harris-II fractures, and distal Salter-Harris IV fractures of the tibia, consistent with findings from Altmann-Schneider et al. for BV [12]. Toddler’s fractures, however, are usually stable under conservative treatment and missed cases have minor clinical impact [18, 19]. “Trampoline fractures”, often presenting as subtle buckle fractures or anterior tilt of the proximal tibial physis, are easily overlooked [20, 21], though their long-term significance – particularly the risk of valgus deformity – remains debated [22]. Distal tibial Salter-Harris-II fractures require accurate detection due to the risk of premature physeal closure and subsequent growth disturbances [23, 24].

### Forearm

All AI tools demonstrated high sensitivity and specificity for fracture detection, with RBf showing superior specificity compared to BV, consistent with prior studies on upper extremity fractures [8, 13, 16]. However, performance varied significantly by fracture types.

Detection of radial and ulnar bowing fractures was markedly low, in line with findings by Altmann-Schneider et al. for BV [12]. Due to the limited number of bowing fractures in prior studies – e.g. Hayashi et al. reported only one case, which AI failed to detect [16] – comparative analysis remains constrained. Bowing fractures are among the most diagnostically challenging in paediatrics, given physiological variation and corresponding higher interrater variability [25]. Many cases are often only recognized in the presence of associated injuries or on follow-up imaging. Although the angulation threshold for reduction remains debated, timely diagnosis is crucial to prevent functional impairment [26].

### Elbow

Performance for elbow fractures was lower than for forearm and lower leg fractures, with medial epicondylar avulsion fractures being particularly challenging. These fractures are difficult to detect due to age-dependent ossification, occasional lack of metaphyseal fragments, and potential joint entrapment of fragments, which can be obscured by other structures [27]. Accurate diagnosis is critical to avoid long-term complications [27, 28].

Several studies have assessed AI for paediatric elbow injuries. Dupuis et al. reported MS sensitivity of 92.9% and specificity of 76.8% across 758 elbow radiographs [29]. Rayvolve (AZmed) showed comparable sensitivity (91.8%) and higher specificity (87.3%) in a cohort of 294 elbow radiographs [13]. For BV, sensitivity ranged 80.5–100% and specificity 84.7–90% across three studies, though fracture prevalence was low [13, 30], and lower performance was reported in a larger dataset [12]. RBf was assessed in one study of 180 paediatric radiographs (sensitivity 89%, specificity 90%) [31], and in a mixed-age cohort, including 98 children (overall 334 patients), Bachmann et al. reported sensitivity of 90% and specificity of 93% [32].

In our study, RBf achieved significantly higher specificity than BV. Busson et al. compared MS, Rayvolve, and BV [33], reporting an overall lower performance for Rayvolve. MS and BV results were comparable to ours, though comparisons were limited by age-selection (≥15 years) and small region-specific samples. Notably, detection of proximal metaphyseal radius and ulna fractures was lower on forearm than elbow radiographs for BV and MS, possibly reflecting differences in model training or fracture prevalence.

Our study assessed indirect fracture signs, as a relevant proportion of elbow fractures are occult and only inferred from a positive fat pad sign indicating elbow effusion [5]. Since occult fractures require the same treatment as visible ones, detection of the fat pad sign is clinically crucial [5, 6]. In our study, AI struggled particularly with the posterior fat pad sign, with MS performing best and BV worst (sensitivity, 40.6%; NPV, 60.2%). A prior study using a deep convolutional neural network reported higher performance (sensitivity 90.0%, specificity 90.6%) [10], though without distinguishing anterior from posterior fat pad and using a small dataset.

Detection of elbow dislocations, including Monteggia fracture-dislocations, was poor for BV and MS (sensitivities 54.2% and 64.6%). RBf detected more cases (sensitivity 93.8%) but with a low PPV (53.6%) and reduced specificity compared to BV and MS. It should be noted that RBf is not approved for use in children <15 years. Overall, none of the tested AI tools demonstrated reliable performance for elbow dislocation detection, though timely recognition is essential to avoid complications [34].

Our study included a detailed analysis of doubt cases, offering insights and guidance for clinicians interpreting uncertain AI-generated reports. To date, only Altmann-Schneider et al. [12] have examined doubt cases in paediatric imaging. BV and RBf produced more uncertain findings than MS, yet the overall PPV of doubt cases was low, indicating that they should generally be interpreted with caution or considered as negative. An exception was the posterior fat pad sign, where doubt cases were predominantly true positives. This underscores both the difficulty of detecting the posterior fat pad and the broader challenge of interpreting doubt cases in clinical practice.

Besides the discussed vendor-specific limitations of the tested AI tools, certain AI-specific limitations must be considered. AI performance strongly depends on the training data set, which varies across all AI tools in terms of patient population and ground truth quality. Furthermore, a lack of transparency concerning algorithm design, training strategies, and generation of doubt cases makes it difficult for clinicians to identify potential sources of error [35, 36].

Our study provides an extensive dataset for three common paediatric fracture sites, enabling detailed analysis of population- and region-specific fracture types and indirect fracture signs. The reference standard was defined by experienced paediatric radiologists with access to clinical information and follow-up studies, ensuring a high-quality ground truth.

Nonetheless, several limitations must be acknowledged. The analysis was restricted to three anatomical sites, limiting generalizability. The retrospective, single-centre design may have introduced bias, and excluding children under 2 years, due to age restrictions in two tested AI tools (BV, RBf) may affect representativeness. Finally, the long inclusion period and variety of sites may have introduced heterogeneity in image quality.

## Conclusion

This study provides important insights into the diagnostic accuracy of three commercially available AI tools for paediatric fracture detection, including indirect signs and joint dislocations. It identifies which posttraumatic changes are reliably detected and highlights areas requiring cautious interpretation or further training. Understanding the strengths and limitations of each AI tool is essential for clinicians to ensure their safe and effective use in paediatric practice.

## Supplementary Information

Below is the link to the electronic supplementary material.

ESM 1 Supplementary Material 1 (DOCX 49.0 KB)

## Data Availability

No datasets were generated or analysed during the current study.
