# Supplementary material for: Multivendor comparison study of artificial intelligence software for automated fracture detection in paediatric patients
Source: Pediatr Radiol. 2025 Nov 18;56(9):2067–77. doi: 10.1007/s00247-025-06461-6 (PMC13546333; doi:10.1007/s00247-025-06461-6)
Supplement: Supplementary file 1 [file 247_2025_6461_MOESM1_ESM.docx]

**Supplementary material**

**Supplementary Material 1 Lower leg comparative analyses**

|  | BV vs. MS | BV vs. RBf | MS vs. RBf | All three AI tools |
| --- | --- | --- | --- | --- |
| Sensitivity | *P*=0.60 (+1.8%) | *P*=0.23 (+3.4%) | *P*=0.60 (+1.6%) | *P*=0.11 |
| Specificity | *P*=0.13 (-0.9%) | *P*=0.80 (+0.2%) | *P*=0.08 (+1.1%) | *P*=0.05 |

*AI* artificial intelligence, *BV* BoneView, *MS* Milvue Suite, *RBf* RBfracture

**Supplementary Material 2 Lower leg fracture types with detection rates**

| Fracture (#) type^a^ | Number of fractures/  frequency (%) | Detection rate (%)/Positive findings^b^ | | |
| --- | --- | --- | --- | --- |
|  |  | BoneView | Milvue Suite | RBfracture |
| Oblique diaphyseal # tibia | 189/29 | 100/189 | 100/189 | 100/189 |
| Metaphyseal buckle # proximal tibia | 60/9 | 83.3/50 | 75.0/45 | 78.3/47 |
| Toddler’s # | 58/9 | 89.7/52 | 82.8/48 | 86.2/50 |
| Transverse diaphyseal # tibia | 38/6 | 100/38 | 100/38 | 97.4/37 |
| Metaphyseal buckle # distal tibia | 36/5 | 100/36 | 97.2/35 | 94.4/34 |
| Transverse diaphyseal # fibula | 31/5 | 93.6/29 | 96.8/30 | 93.6/29 |
| Oblique diaphyseal # fibula | 31/5 | 90.3/28 | 90.3/28 | 83.9/26 |
| Complete metaphyseal # distal fibula | 30/4 | 100/30 | 100/30 | 90.0/27 |
| Salter-Harris II # distal tibia | 29/4 | 82.8/24 | 82.8/24 | 86.2/25 |
| Metaphyseal buckle # distal fibula | 26/4 | 80.8/21 | 80.8/21 | 69.2/18 |
| Complete metaphyseal # distal tibia | 25/4 | 100/25 | 100/25 | 100/25 |
| Complete metaphyseal # proximal fibula | 19/3 | 84.2/16 | 94.7/18 | 94.7/18 |
| Complete metaphyseal # proximal tibia | 18/3 | 100/18 | 100/18 | 100/18 |
| Salter-Harris II # proximal tibia | 15/2 | 80/12 | 80/12 | 73.3/11 |
| Salter-Harris IV # distal tibia | 13/2 | 84.6/11 | 84.6/11 | 76.9/10 |
| Metaphyseal buckle # proximal fibula | 10/1 | 100/10 | 100/10 | 80/8 |

^a^ Displayed if ≥10 cases detected

^b^ True positive findings and doubt cases

**Supplementary Material 3 Forearm comparative analyses**

|  | BV vs. MS | BV vs. RBf | MS vs. RBf | All three AI tools |
| --- | --- | --- | --- | --- |
| Sensitivity | *P*=0.44 (-1.2%) | *P*=0.54 (+0.6) | *P*=0.16 (+1.8%) | *P*=0.13 |
| Specificity | *P*=0.008 (-2.8%) | *P*=0.004 (-3.1%) | *P*=0.82 (-0.3%) | *P*<0.001 |

*AI* artificial intelligence, *BV* BoneView, *MS* Milvue Suite, *RBf* RBfracture

**Supplementary Material 4 Forearm fracture types with detection rates**

| Fracture (#) type^a^ | Number of fractures/  frequency (%) | Detection rate (%)/Positive findings^b^ | | |
| --- | --- | --- | --- | --- |
|  |  | BoneView | Milvue Suite | RBfracture |
| Metaphyseal buckle # distal radius | 326/24 | 99.4/324 | 99.1/323 | 98.5/320 |
| Metaphyseal buckle # distal ulna | 204/15 | 87.7/179 | 90.7/185 | 84.8/173 |
| Complete metaphyseal # distal radius | 117/9 | 100/117 | 100/117 | 100/117 |
| Transverse diaphyseal # radius | 104/8 | 100/104 | 100/104 | 100/104 |
| Complete metaphyseal # distal ulna | 72/5 | 98.6/71 | 100/72 | 100/72 |
| Greenstick # radius | 70/5 | 100/70 | 100/70 | 98.6/69 |
| Transverse diaphyseal # ulna | 63/5 | 100/63 | 100/63 | 100/63 |
| Greenstick # ulna | 61/5 | 95.1/58 | 98.4/60 | 95.1/58 |
| Oblique diaphyseal # ulna | 55/4 | 100/55 | 100/55 | 100/55 |
| Salter-Harris II # distal radius | 47/3 | 100/47 | 100/47 | 97.9/46 |
| Oblique diaphyseal # radius | 38/3 | 100/38 | 97.4/37 | 100/38 |
| Avulsion # of the styloid process ulna | 33/2 | 84.8/28 | 97.0/32 | 78.8/26 |
| Metaphyseal buckle # proximal radius | 26/2 | 80.8/21 | 84.6/22 | 84.6/22 |
| Bowing # ulna | 22/2 | 18.2/4 | 13.6/3 | 22.7/5 |
| Bowing # radius | 19/1 | 10.5/2 | 21.1/4 | 10.5/2 |
| Complete metaphyseal # proximal ulna | 18/1 | 83.3/15 | 83.3/15 | 83.3/15 |
| Complete supracondylar # humerus | 17/1 | 94.1/16 | 100/17 | 94.1/16 |
| Salter-Harris II # proximal radius | 12/1 | 83.3/10 | 91.7/11 | 91.7/11 |
| Complete diaphyseal # ulna (Monteggia) | 11 | 100/11 | 100/11 | 100/11 |

^a^ Displayed if ≥10 cases detected

^b^ True positive findings and doubt cases

**Supplementary Material 5 Elbow comparative analyses**

|  | BV vs. MS | BV vs. RBf | MS vs. RBf | All three AI tools |
| --- | --- | --- | --- | --- |
| *Fractures* |  |  |  |  |
| Sensitivity | *P*=0.63 (-0.9%) | *P*=0.15 (+3.2%) | *P*=0.06 (+4.1%) | *P*=0.03 |
| Specificity | *P*=0.33 (-2.14%) | *P*=0.004 (-6.5%) | *P*=0.05 (-4.4%) | *P*=0.004 |
| *Dislocations* |  |  |  |  |
| Sensitivity | *P*=0.41 (-10.4%) | *P*<0.001 (-39.6%) | *P*=0.002 (-29.2%) | *P*<0.001 |
| Specificity | *P*<0.001 (+0.8%) | *P*<0.001 (+3.0%) | *P*<0.001 (+2.2%) | *P*<0.001 |
| *Effusion, anterior* |  |  |  |  |
| Sensitivity | *P*=1 (-0.2%) | *P*=1 (-0.2%) | *P*=1 (+0.0%) | *P*=0.10 |
| Specificity | *P*<0.001 (-5.9%) | *P*<0.001 (+8.2%) | *P*<0.001 (+14.1%) | *P*<0.001 |
| *Effusion, posterior* |  |  |  |  |
| Sensitivity | *P*<0.001 (-41.7%) | *P*<0.001 (-33.0%) | *P*<0.001 (+8.7%) | *P*<0.001 |
| Specificity | *P*=0.21 (-1.3%) | *P*=0.04 (-1.8%) | *P*=0.45 (-0.5%) | *P*=0.02 |

*AI* artificial intelligence, *BV* BoneView, *MS* Milvue Suite, *RBf* RBfracture

**Supplementary Material 6 Elbow fracture types with detection rates**

| Fracture (#) type^a^ | Number of fractures/  frequency (%) | Detection rate (%)/Positive findings^b^ | | |
| --- | --- | --- | --- | --- |
|  |  | BoneView | Milvue Suite | RBfracture |
| Complete supracondylar # humerus | 293/45 | 99.3/291 | 99.0/290 | 98.3/288 |
| Supracondylar buckle # humerus | 71/11 | 93.0/66 | 84.5/60 | 57.7/41 |
| Salter-Harris IV # radial condyle | 65/10 | 95.4/62 | 89.2/58 | 90.8/59 |
| Complete metaphyseal # proximal ulna | 50/8 | 80/40 | 90/45 | 80/40 |
| Salter-Harris II # proximal radius | 44/7 | 95.5/42 | 95.5/42 | 97.7/43 |
| Metaphyseal buckle # proximal radius | 32/5 | 91.9/23 | 81.3/30 | 84.4/27 |
| Avulsion # medial epicondyle | 22/3 | 36.4/8 | 63.6/14 | 40.9/9 |
| Complete metaphyseal # proximal radius | 20/3 | 100/20 | 100/20 | 100/20 |
| Salter-Harris II # ulnar condyle | 10/2 | 90/9 | 90/9 | 100/10 |

^a^ Displayed if ≥10 cases detected

^b^ True positive findings and doubt cases

**Supplementary Material 7 Elbow effusion doubt cases**

|  | Milvue Suite | | RBfracture | |
| --- | --- | --- | --- | --- |
|  | Anterior effusion | Posterior effusion | Anterior effusion | Posterior effusion |
| True positive/total doubt, *n* | 34/38 | 48/51 | 68/122 | 53/53 |
| PPV of doubt cases | 89.5 (75.9–95.8) | 94.1 (84.1–98.0) | 55.7 (46.9–64.2) | 100 (93.2–100) |

*n* number
